# Transverse abdominis plane block compared with patient-controlled epidural analgesia following abdominal surgery: a meta-analysis and trial sequential analysis

**DOI:** 10.1038/s41598-022-25073-w

**Published:** 2022-11-29

**Authors:** Young Hyun Jeong, Ji-Yoon Jung, Hyeyeon Cho, Hyun-Kyu Yoon, Seong-Mi Yang, Ho-Jin Lee, Won Ho Kim

**Affiliations:** Department of Anesthesiology and Pain Medicine, Seoul National University Hospital, Seoul National Unversity College of Medicine, 101 Daehak-Ro, Jongno-Gu, Seoul, 03080 Republic of Korea

**Keywords:** Outcomes research, Pain

## Abstract

Thoracic epidural analgesia (TEA) and transversus abdominis plane (TAP) block are used for pain control after abdominal surgery. Although there have been several meta-analyses comparing these two techniques, the conclusion was limited by a small number of studies and heterogeneity among studies. Our meta-analysis used the Medline, EMBASE, and Cochrane central library databases from their inception through September 2022. Randomized controlled trials (RCTs) comparing TEA and TAP block were included. The pre-specified primary outcome was the pain score at rest at 12 h postoperatively. Twenty-two RCTs involving 1975 patients were included. Pooled analyses showed the pain score at rest at 12 h postoperatively was significantly different between groups favoring TEA group (Mean difference [MD] 0.58, 95% confidence interval CI − 0.01, 1.15, P = 0.04, I^2^ = 94%). TEA group significantly reduced the pain score at 48 h at rest (MD 0.59, 95% CI 0.15, 1.03, P = 0.009, I^2^ = 86%) and at 48 h at movement (MD 0.53, 95% CI 0.07, 0.99, P = 0.03, I^2^ = 76%). However, there was no significant difference at other time points. Time to ambulation was shorter in TAP block but the incidence of hypotension at 24 h and 72 h was significantly lower in TAP block compared to TEA. Trial sequential analysis showed that the required information size has not yet been reached. Our meta-analysis demonstrated there was no significant or clinically meaningful difference in the postoperative pain scores between TEA and TAP block group. Given the insufficient information size revealed by TSA, the high risk of bias of our included studies, and the significant heterogeneity of our meta-analysis results, our results should be interpreted carefully but it is not likely that the addition of further studies could prove any clinically meaningful difference in pain score between these two techniques.

## Introduction

Postoperative pain control has been important for patient satisfaction, lower complication rates, shorter hospital stays, and lower medical costs. In addition, with the recent emergence of the concept of enhanced recovery after surgery, interest in early recovery has grown, and multimodal and active control of pain in patients is becoming increasingly important.

Previously, it was recommended to perform thoracic epidural analgesia (TEA) for analgesia in patients who underwent abdominal surgery, but epidural analgesia has various risks such as catheter failure, hypotension, and epidural hematoma. As transversus abdominis plane (TAP) blocks appeared, they are increasingly being actively used. TAP block is to block the thoracoabdominal nerves by injecting drugs or installing a catheter under ultrasound-guided or direct vision.

So far, there have been several meta-analyses comparing TEA and TAP blocks after abdominal surgery^1–3^. Previous meta-analyses showed that there was no significant difference in postoperative pain scores. However, previous meta-analyses found it difficult to draw a definitive conclusion due to the limitations regarding the small number of studies, the small number of total participants, and the large heterogeneity among studies.

The purpose of our meta-analysis is to provide an updated analysis to compare the analgesic effect, functional outcomes, and side effects of TEA and TAP blocks in patients who underwent open or laparoscopic abdominal surgery under general anesthesia. Accordingly, we collected prospective randomized controlled trials (RCTs) and performed a meta-analysis, systematic review, and trial sequential analysis.

## Methods

The current systematic review with meta-analysis to compare TAP block with TEA was conducted according to the recommendations of the Cochrane Handbook for Systematic Reviews of Interventions^4^ and was reported according to the Preferred Reporting Items for Systematic Reviews and Meta-Analyses (PRISMA) statements^5^. The protocol was registered on PROSPERO (registration number: CRD42021241020). There were no deviations from the pre-registered protocol. We carried out a systematic search of the Medline, Embase and the Cochrane Central Register of Controlled Clinical Trials from inception to December 18, 2021. The search was updated on September 2022 during the manuscript revision process. The search strategy of Medline was (Epidural anaesthesia OR Epidural anesthesia OR Caudal anaesthesia OR Caudal anesthesia OR Epidural injection OR Epidural drug administration OR Epidural analgesia) AND (Abdominal wall block OR Abdominal wall injection OR Abdominal wall analgesia OR Abdominal wall anesthesia OR Transversus Abdominal wall block OR Transversus abdominis plane block OR Transversalis abdominis block OR Transverse abdominal plane block OR TAP block). We included only randomized controlled trials, which were published in the English language. Randomized clinical trials comparing TAP block with TEA in adult patients undergoing open or laparoscopic abdominal surgery under general anesthesia were included. Two authors (YHJ and WHK) independently screened the search results using the title and abstract. The full texts of potentially eligible articles were evaluated for their inclusion. We used only Review Manager (RevMan version 5.3. Copenhagen: The Nordic Cochrane Centre, The Cochrane Collaboration, Oxford, United Kingdom) to select the studies and did not use any other reference manager software.

After determining all included studies, the risk of bias in individual studies was evaluated using the bias domains described in the Cochrane Handbook for Systematic Reviews of Interventions, version 5.1.0.^6^ including the following domains: allocation concealment (selection bias), random sequence generation (selection bias), incomplete outcome data (attrition bias), blinding of participants and personnel (performance bias), selective reporting (reporting bias), and other sources of bias (other bias). Disagreements were resolved by discussion between the two authors or, if needed, by the involvement of another author.

The level of certainty of the evidence for all our study outcomes was determined using the Grading of Recommendations, Assessment, Development, and Evaluation (GRADE) system, which consists of five domains: risk of bias, inconsistency, indirectness, imprecision, and publication bias^7^.

Data including inclusion and exclusion criteria, sample size, the technique of TAP block (method of localization, unilateral or bilateral, site of injection, single shot or continuous catheter technique, type of local anesthetics, or TEA (method of localization, type of local anesthetic, bolus and infusion protocol) and postoperative analgesia regimen were collected by one author (YHJ), the accuracy of which was confirmed by another author (WHK).

The primary outcomes were the pain score at rest at 12 h postoperatively, which was scored on a 0–10 numerical rating scale (NRS). The secondary outcomes were the postoperative pain score at rest at 0–2 h, 24 h, 48 h and 72 h, and the postoperative pain score on movement at 0–2 h, 12 h, 24 h. 48 h and 72 h. The following outcomes were also included; the total opioid consumption (converted to IV-morphine equivalent); failure rate; incidence of postoperative nausea and vomiting (PONV); incidence of hypotension at 24 h and 72 h.

### Statistical analysis

We conducted analyses using Review Manager (RevMan version 5.3. Copenhagen: The Nordic Cochrane Centre, The Cochrane Collaboration, Oxford, United Kingdom).

Continuous variables were extracted as mean and standard deviations. If trials reported continuous variables as median and interquartile range, the mean was assumed to be equivalent to the median and the standard deviation was estimated to be the interquartile range divided by 1.35^4^. We used a random-effects model (inverse variance method for a continuous outcome and Mantel–Haenszel method for a dichotomous outcome) to approximate the effect size of outcome variables. We presented the effect size as a pooled odds ratio (OR), pooled mean difference (MD) with a 95% confidence interval (CI), and depicted a forest plot.

Statistical heterogeneity was assessed by the coefficient I^2^. We graded heterogeneity according to predetermined thresholds for high (≥ 75%), moderate (50–74%), and low (25–49%) levels^8,9^. We assessed publication bias by drawing and visually examining a funnel plot. Duval and Tweedie’s trim and fill test and Egger’s linear regression test were also used to evaluate the publication bias using Stata/SE version 13.0 (StataCorp, College Station, TX, USA).

We conducted a trial sequential analysis (TSA) with TSA Viewer (Version 0.9.5.10 Beta, Copenhagen Trial Unit, 2016, Copenhagen, Denmark)^10^. All studies in the three subgroups of open, laparoscopic, and combined surgery were included for each TSA. TSA conducts a cumulative meta-analysis, which depicts a Z curve of the pooled observed effect using the cumulative number of participants and events. TSA constructs two different boundaries for preference for intervention or control group or futility – a conventional boundary for conventional significance (P < 0.05) and the trial sequential boundary (O’Brien–Fleming significance boundary). TSA also provides the required information size which means the sufficient sample size required to confirm or reject a certain effect of the study intervention. The required information size was estimated with an 80% power and alpha error of 5%. We depicted two-sided 5% symmetrical O’Brien–Fleming significance boundaries as well as a conventional boundary.

## Results

**Figure 1:**
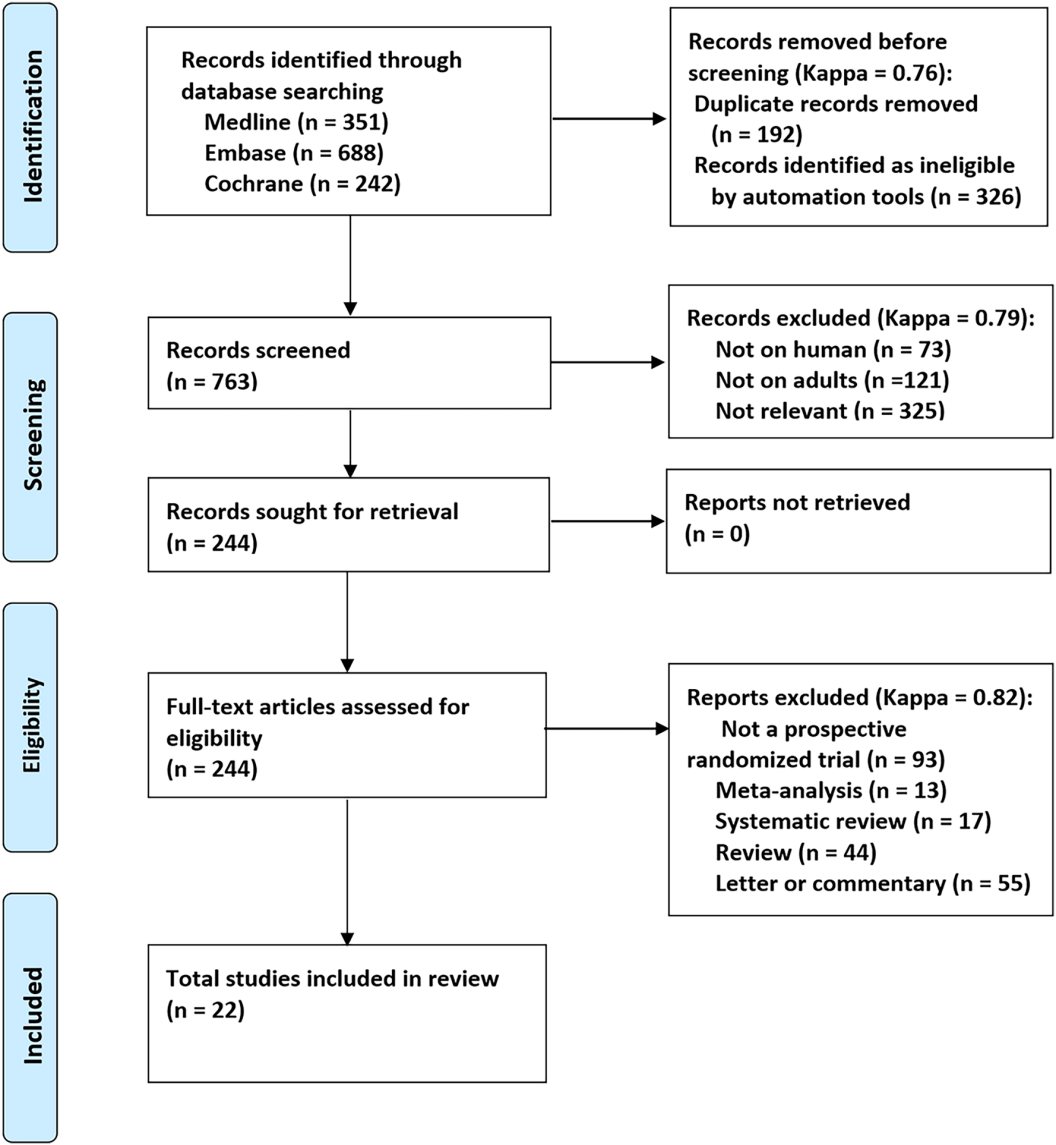
PRISMA (Preferred reporting times for systematic reviews and meta-analyses) 2020 flow diagram.

A total of 1281 publications were identified according to our search strategy. After screening 1281 titles and abstracts, 192 duplicate studies and 326 irrelevant studies were excluded. Finally, 22 RCTs were included after carefully reviewing the full text. Figure 1 shows details of the screening and exclusion process.

The baseline characteristics of our included randomized trials are summarized in Table 1. Studies were published between 2011 and 2022. A total of 1975 patients participated, of which 997 were in the TAP block group, and 978 of them were in the epidural group. Of the 22 studies^11–32^, 3 studies were subjected to the patients who underwent laparoscopic surgery^11,22,32^, 15 studies were to open surgery^12–14,16–21,23,24,26,27,30,31^ and 4 studies subjected to both types of surgery^15,25,28,29^.

**Table 1:** Characteristic of the included trials.

Trial	Group- TAP block	Group-Epidural	Surgery	TAP block technique	Local anesthetic for TAP block	Local anesthetic for epidural	Postoperative analgesia
Aditianingsih et 2018^11^	25	25	Laparoscopic donor nephrectomy with a Pfannenstiel incision	USG, bilateral lateral and unilateral subcostal approach, single-shot injection	0.25% bupivacaine 20 ml for each injection	Single bolus of 0.125% bupivacaine 3 ml and continuous infusion of 0.125% bupivacaine at a rate of 6 ml/h	IV morphine PCA
Calixto-Flores 2020^12^	15	15	Open donor nephroureterectomy	Surgical placement under direct vision, unilateral lateral approach, continuous block	0.375% ropivacaine 15 ml bolus injection and continuous infusion of 0.2% ropivacaine at a rate of 2 ml/h	Single bolus of 0.375% ropivacaine 10 ml and continuous infusion of 0.2% ropivacaine at a rate of 2 ml/h	Not described
Canakci 2018^13^	42	42	Cesarean section	USG, bilateral lateral approach, sigle shot injection	0.25% bupivacaine 20 ml for each injection	Single bolus of 0.5% isobaric bupivacaine 16 ml, morphine 3 ml, and fentanyl 50 mcg (20 ml in total)	Intravenous dexketoprofen
Cata 2021^14^	35	33	Cytoreductive surgery with hyperthermic intraperitoneal chemotherapy	USG, bilateral lateral and subcostal approach, single shot injection	Bupivacaine 150 mg and liposomal bupivacaine 266 mg divided into four quadrants	bupivacaine 0.075% ± hydromorphone 2–5 mcg/mL or bupivacaine 0.075% ± fentanyl 5 mcg/mL, basal rate 8 mL/h, bolus 3 mL every 10 min	Regular paracetamol, oral nonopioid analgesics (ex. Celecoxib) PRN
Felling 2018^15^	92	87	Open, laparoscopic and robotic abdominal surgery	USG, bilateral lateral approach, single-shot injection	133 mg liposomal bupivacaine 20 ml on each side	Continuous infusion of 0.0625% bupivacaine and fentanyl of unspecified concentration at rate of 6–8 ml/h	Regular paracetamol, naproxen and gabapentin
Ganapathy 2015^16^	26	24	Laparotomy	USG, bilateral lateral and subcostal approach, continuous block	1. Lateral TAP: 10 ml ropivacaine 0.5% bolus injection on each side	0.25% bupivacaine 5 ml ± additional 0.25% bupivacaine 3 ml boluses followed by a continuous postoperative infusion of 0.1% bupivacaine and hydromorphone 10 mcg/ml at a rate of 8 ml/h for 72 h	Regular paracetamol, naproxen and gabapentin
					2. Subcostal TAP: 20 ml ropivacaine 0.5% bolus injection on each side		
					3. Single lateral and subcostal TAP injections followed by a combined continuous infusion of ropivacaine 0.35% at a rate of 4–5 ml/h on each side for 72 h		
Hughes 2015^17^	49	44	Open liver surgery	Surgical placement under direct vision, unilateral lateral and rectus sheath approaches, continuous block	40 ml levobupivacaine 0.125% bolus injection in total followed by a combined continuous injection of levobupivacaine 0.375% at a rate of 4 ml/h for 48 h	10 ml levobupivacaine of unspecified concentration followed by a continuous infusion of 0.1% levobupivacaine at an unspecified rate	IV morphine PCA
Kandi 2015^18^	30	30	Laparotomy	USG, bilateral lateral approach, single-shot injection	20 ml bupivacaine 0.125% on each side	Continuous infusion of 0.125% bupivacaine at a rate of 4–8 ml/h for 48 h unless still needed for pain relief	Paracetamol and morphine PRN
Lyer 2017^19^	33	36	Open lower abdominal surgery	USG, bilateral lateral approach, just above the iliac crest, single-shot injection and subsequent top-ups at 8 hourly intervals for 48 h	20 ml 0.125% bupivacaine on each side for the first bolus and subsequent top-ups of the same volume and concentration at 8 hourly intervals for 48 h	First dose at the end of surgery – 0.125% bupivacaine 10 ml and subsequent top-ups of the same volume and concentrations at 8 hourly intervals for 48 h	Regular paracetamol and IV tramadol
Mathew 2019^20^	20	20	Total abdominal hysterectomy with a Pfannenstiel incision	Landmark-guided bilateral lateral approach, single-shot injection	15 ml bupivacaine 0.25% on each side	1. Intraoperative: 2% lidocaine 6–8 ml with epinephrine 5 mcg/ml every 90 min	Morphine PRN
						2. Postoperative: 0.125% bupivacaine 8 ml every 6 h for 24 h	
Niraj 2011^21^	27	31	Laparotomy	USG, bilateral subcostal approach, continuous block	1 mg/kg bupivacaine 0.375% boluses every 8 h through each catheter for 72 h	0.25% bupivacaine 20 ml followed by a continuous postoperative infusion of 0.125% bupivacaine and fentanyl 2 mcg/ml at a rate of 6–12 ml/h and a bolus of 2 ml with a lockout period of 30 min for 72 h	Regular paracetamol and IV tramadol, epidural analgesia if TAP block failed and IV morphine PCA if epidural failed
Niraj 2014^22^	30	31	Laparoscopic abdominal surgery	USG, bilateral lateral approach, continuous block and bilateral subcostal approach, single-shot injection	0.375% levobupivacaine 2.5 ml/kg in total for all four quadrant blocks followed by a continuous infusion of 0.25% levobupivacaine through both catheters at a rate of 8–10 ml/h for 48 h	0.25% bupivacaine 20 ml followed by a continuous infusion of 0.125% bupivacaine and fentanyl 2 mcg/ml at a rate of 8–12 ml/h and a bolus of 2 ml with a lockout period of 30 min	Regular paracetamol and diclofenac with tramadol PRN
Raghvendra 2016^23^	30	30	Total abdominal hysterectomy	USG, bilateral lateral approach, single-shot injection	0.75% ropivacaine 1.5 ml/kg at a maximum dose of 150 mg on each side	0.5% ropivacaine 10–15 ml ± additional 0.5% ropivacaine 5 ml bolus to reach a sensory block up to T8 followed by a continuous postoperative infusion of 0.2% ropivacaine at a rate of 10 ml/h	IV tramadol PCA
Rao Kadam 2013^24^	22	19	Laparotomy	USG, bilateral lateral or subcostal approach depending on the surgery, continuous block	0.375% ropivacaine 20 ml bolus injection each side followed by a continuous infusion of	0.2% ropivacaine 8–15 ml followed by a continuous postoperative infusion of 0.2% ropivacaine at a rate of 5–15 ml/h for 72 h	Regular paracetamol and IV fentanyl PCA
Regmi 2019^25^	35	35	Lower abdominal surgery	USG, bilateral lateral approach, continuous block	0.25% bupivacaine 0.4 ml/kg at a maximum dose of 2 mg/kg on each side followed by a continuous infusion of 0.125% bupivacaine at a rate of 5 ml/h through each catheter for 24 h	0.25% bupivacaine 15 ml followed by a continuous postoperative infusion of 0.125% bupivacaine at a rate of 5–12 ml/h for 24 h	IV morphine PCA
Revie 2012^26^	49	44	Open liver surgery	Surgical placement under direct vision, unilateral lateral and rectus sheath approaches, continuous block	0.25% levobupivacaine 20 ml bolus injection	Continuous infusion of 0.1% bupivacaine and fentanyl 2 mcg/ml at a rate of 7–10 ml/h	Regular paracetamol for all patients and unspecified opiate PCA in TAP group
Shaker 2018^27^	32	35	Laparotomy	USG, bilateral lateral and subcostal	Liposomal bupivacaine 10 ml and 0.5% bupivacaine on each side	0.125% bupivacaine and fentanyl 2 mcg/ml at an unspecified rate	Paracetamol, ketorolac, gabapentin and opioid PRN
Torgeson 2018^28^	41	37	Laparoscopic or open colorectal surgery	USG, bilateral, subcostal approach, single-shot injection	Liposomal bupivacaine 40 ml (133 mg) on each side	Boluses of bupivacaine 0.0625% and fentanyl 2 mcg/ml intraoperatively followed by continuous postoperative infusion at a rate of 6 ml/h and a bolus of 2 ml with a lock out period of 30 min for 48 h	Regular paracetamol and ketorolac
Turan et al.^29^	260	254	open or laparoscopic-assisted abdominal surgery, including colorectal procedures and hysterectomies	USG, bilateral lateral and subcostal	0.25% bupivacaine 10 ml and liposomal bupivacaine 5 ml (266 mg) on each side	Bolus of bupivacaine 0.1% and patient-controlled boluses allowed per hospital policy (usually 3 ml each, every 15 min)	IV hydromorphone or fentanyl, IV PCA
Wahba 2014^30^	22	22	Laparotomy	USG, bilateral subcostal approach, continuous block	0.25% bupivacaine 20 ml on each side followed by boluses of 0.25% bupivacaine 15 ml every 8 h through each catheter	0.125% bupivacaine 10 ml followed by a continuous postoperative infusion of 0.125% bupivacaine at a rate of 6–8 ml/h	IV morphine PCA
Wu 2013^31^	27	29	Laparotomy	USG, bilateral subcostal approach, single-shot injection	0.375% ropivacaine 20 ml on each side	Before anesthesia induction: 0.25% ropivacaine 8 ml	IV morphine PCA
						Intraoperative: Continuous infusion of ropivacaine	
Xu 2020^32^	55	55	Laparoscopic colorectal cancer surgery	USG, bilateral lateral and subcostal approach, continuous block	0.375% levobupivacaine 2.5 ml/kg in total for all four quadrant blocks followed by a continuous infusion of 0.25% levobupivacaine through both catheters at a rate of 8 ml/h for 48 h	Before anesthesia induction: 0.25% ropivacaine 6–8 ml at least 20 min	Regular flurbiprofen, sufentanil PRN
						Intraoperative: 0.25% ropivacaine 5 ml/h	
						Postoperative: 0.15% ropivacaine and 0.5 μg/ml sufentanil at a continuous infusion rate of 4 ml/h, 3 ml bolus on patient request and 15 min lock-out time, for 48 h	

**Figure 2:**
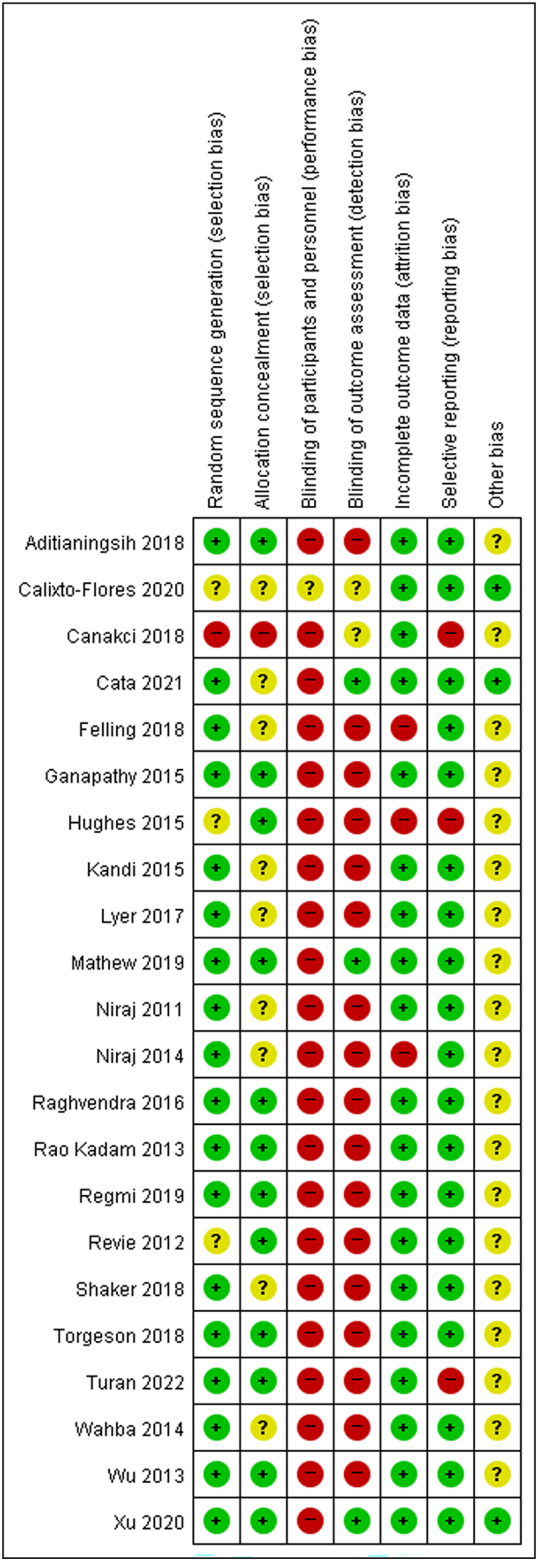
Summary of risk of bias assessment.

The risk of bias assessment is shown in Fig. 2. Most of the studies were evaluated as at a high risk of performance bias and detection bias, due to not performing adequate blinding of participants, personnel, or the outcome assessor.

**Figure 3:**
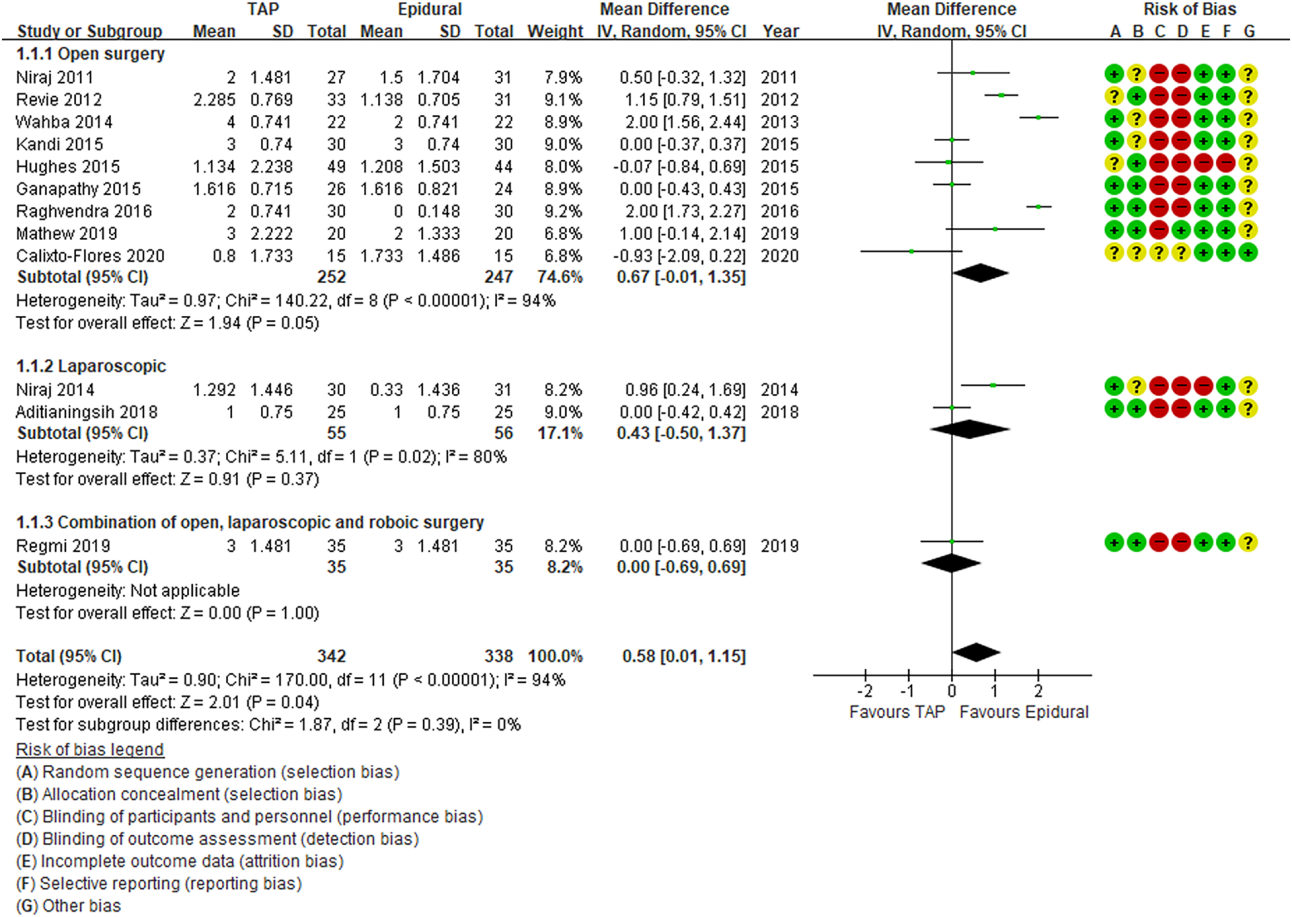
Forest plot of comparison: Pain score at rest at 12 h after surgery.

**Figure 4:**
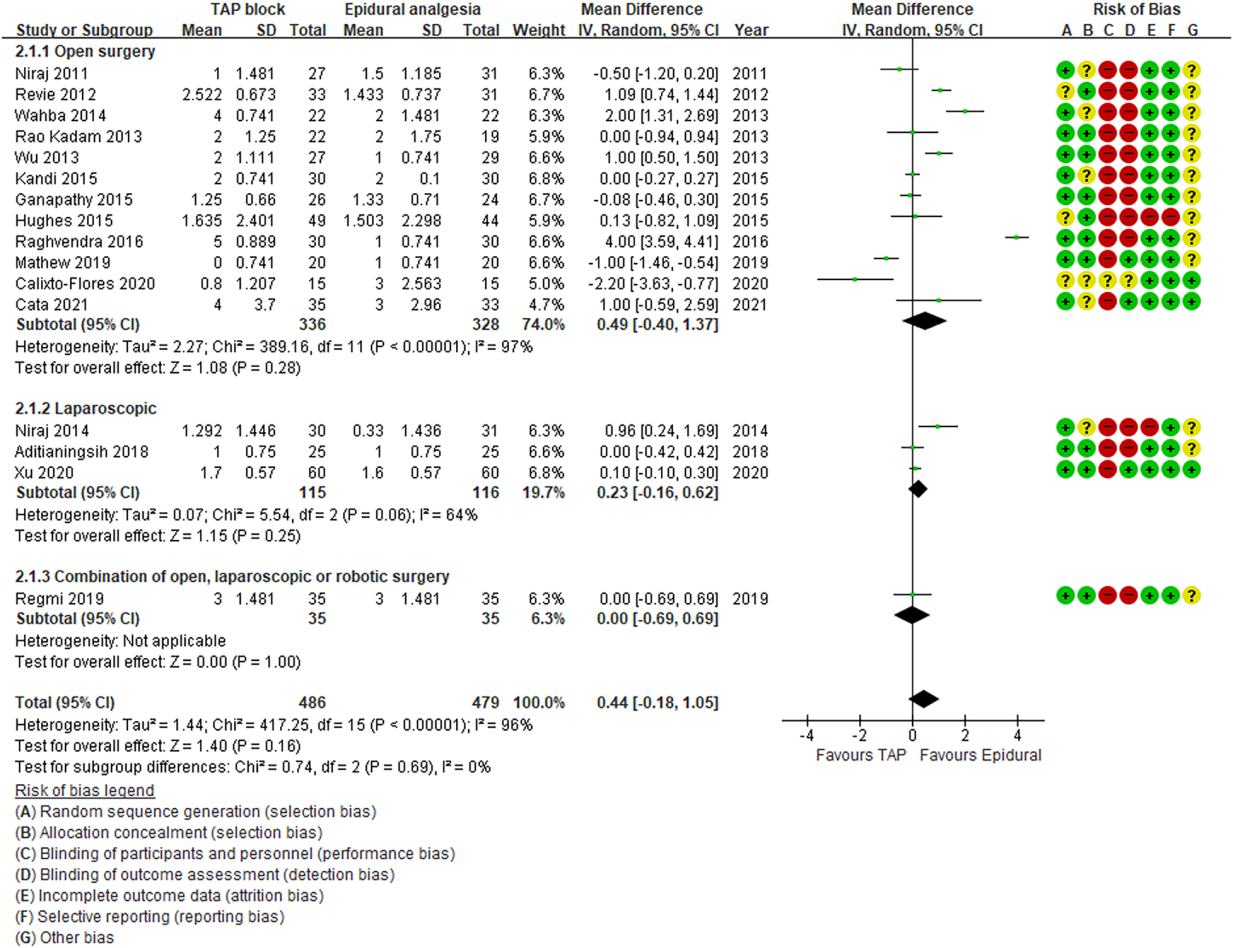
Forest plot of comparison: Pain score at rest at 24 h after surgery.

All studies used the same scale for pain assessment (visual analogue scale 0–10). Our primary outcome of the pain score at rest at 12 h after surgery was significantly different between the TAP block and TEA group favoring TEA group. (MD 0.58, 95% CI 0.01, 1.15, P = 0.04, Fig. 3), with significant heterogeneity (I^2^ = 94%, P < 0.01). The pain score at rest at 24 h was also not significantly different between the two groups. (MD 0.44, 95% CI − 0.18, 1.05, P = 0.16, Fig. 4) with high heterogeneity (I^2^ = 96%, P < 0.01).

**Figure 5:**
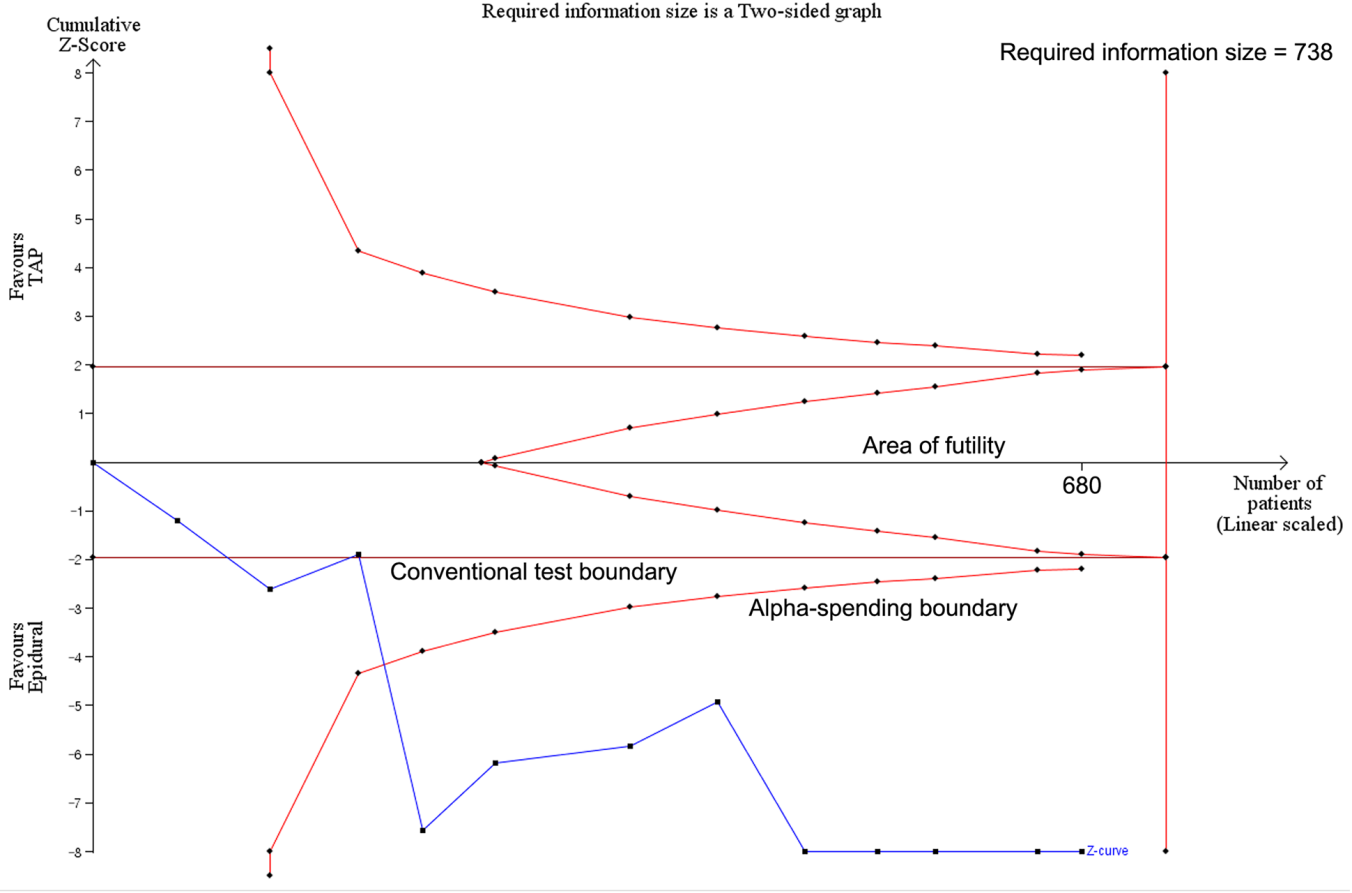
Trial sequential analysis for the pain score at rest at 12 h.

TSA showed the cumulative observed effect of z-curve for postoperative pain score at rest at 12 h exceeded both the conventional boundary and the O’Brien-Fleming significance boundary and remained outside of both boundaries (Fig. 5). This means postoperative pain score at rest at 12 h was significantly lower in the TEA group. However, the number of patients did not surpass the required sample size for this outcome.

**Figure 6:**
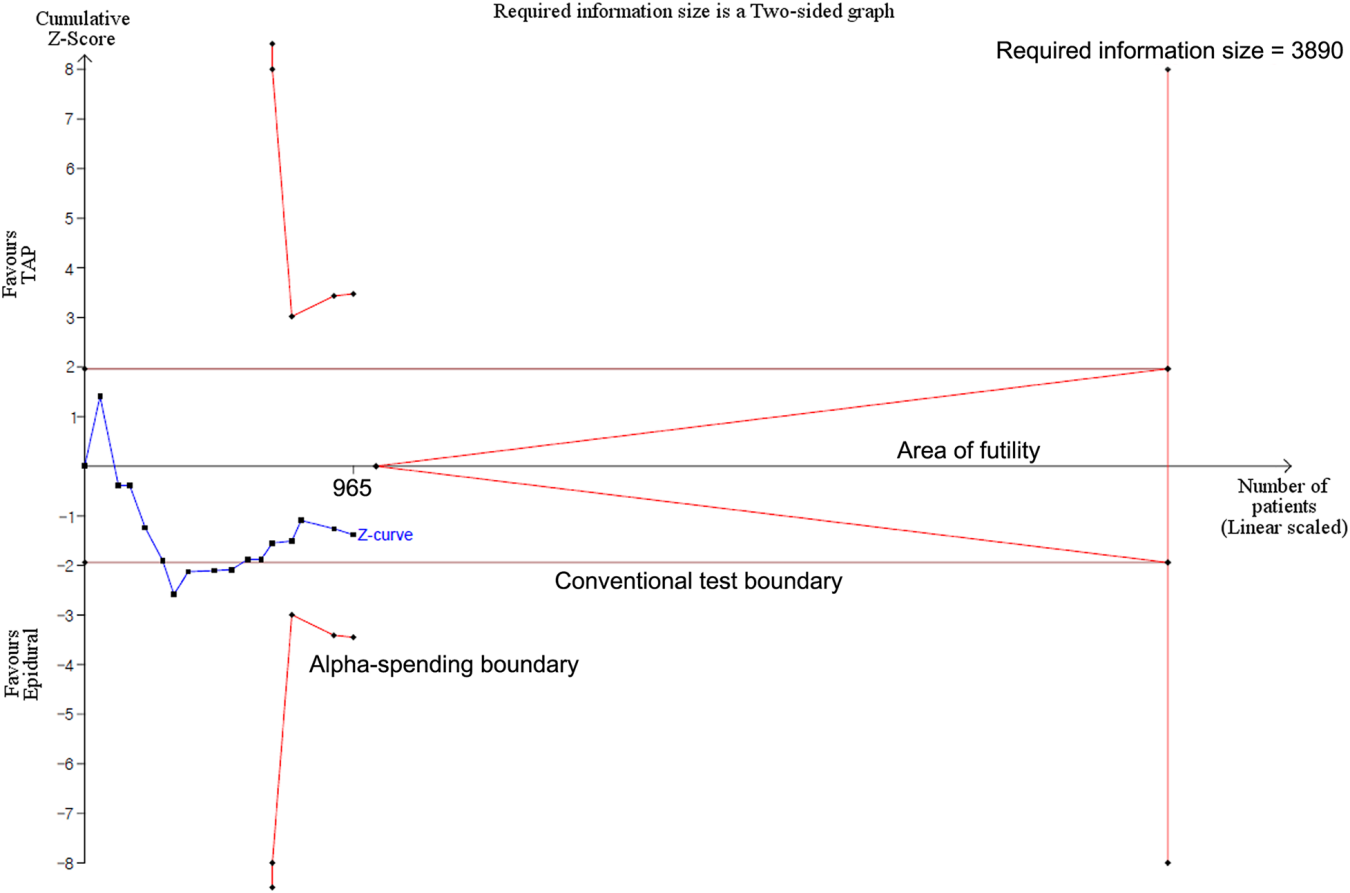
Trial sequential analysis for the pain score at rest at 24 h.

Meanwhile, the cumulative z-curve of postoperative pain score at rest at 24 h did not cross any of the two boundaries, which means that the pain score at 24 h does not significantly differ between the two groups. However, as the cumulative z-score did not enter the area of futility and the required information size was not achieved (Fig. 6).

Funnel plots of our primary outcomes illustrate some symmetric properties, suggesting the absence of publication bias (Supplementary Figs. S1–S2). However, the trim and fill test (P < 0.001) and the Egger’s test (P = 0.031) showed the presence of publication bias.

The results of the meta-analyses of our secondary outcomes were summarized in Supplementary Table S1. TEA group reduced the postoperative pain score at rest at 48 h (MD 0.59, 95% CI 0.15, 1.03, P = 0.009, I^2^ = 86%) and pain score on movement at 48 h (MD 0.53, 95% CI 0.07, 0.99, P = 0.03, I^2^ = 76%). Interval morphine equivalent consumption at each time band (0–24 h, 24–48 h, 48–72 h) was similar between the two groups. Functional outcomes of the time to first flatus and hospital length of stay did not significantly differ between groups. However, time to ambulation (MD − 4.52 h, 95% CI − 8.68, − 0.36, P = 0.03, I^2^ = 70%) was significantly shorter in the TAP block group compared to the TEA group. Regarding complication rates, the failure rate of the procedure was not significantly different between groups. There was no significant difference in the rate of postoperative nausea and vomiting (PONV) between groups (OR = 0.81, 95% CI 0.39, 1.65, P = 0.55, I^2^ = 50%). However, the incidences of hypotension at 24 h and 72 h were significantly higher in the TEA group.

The quality of evidence evaluated with the GRADE system was reported for all primary and secondary outcomes in Supplementary Table S2.

## Discussions

In this meta-analysis, we sought to compare the clinical effect and safety of TEA and TAP block as postoperative analgesia in abdominal surgery under general anesthesia. Meta-analysis and TSA were performed based on the 22 prospective RCTs. Our pooled analysis showed that most of the pain scores were not significantly different between groups. Only the pain scores at 48 h showed statistical significance but the absolute difference was not clinically meaningful. TSA showed the required sample sizes for the pain scores at rest at 12 and 24 h were not achieved, suggesting that further RCTs are required for confirm conclusion. However, time to ambulation and the incidence of hypotension at 24 h and 72 h were significantly different favoring the TAP block group. Our results should be interpreted carefully given the insufficient information size demonstrated by TSA, high risk of bias of the individual studies, significant heterogeneity, and low or very low quality of evidence for most of our outcomes.

According to the results of our meta-analysis, we found no significant difference between TEA and TAP block in both postoperative pain scores at rest at 12 h and 24 h. Additionally, we performed TSA to better control type-1 and type-2 errors. According to the adjusted threshold for statistical significance in TSA, TEA showed a lower score than TAP block in postoperative pain scores at 12 h but not at 24 h. However, for both outcomes, the cumulative number of participants did not reach the required information size. Given the results of insignificant results of our meta-analysis for postoperative pain scores and significant heterogeneity, we can not simply accept the results of TSA for the pain score at 12 h. We think that both TEA and TAP block are effective to control the pain scores and the results of TSA suggest that no conclusion could be drawn until sufficient information size was obtained.

Our meta-analysis of pain scores at other time points showed that there is a significant difference for both pain scores at rest at 48 h and pain scores at movement at 48 h. However, the differences were only 0.59 and 0.53 on the numerical rating scale from 0 to 10 for the pain score at rest and on movement, respectively. We think that these small differences in pain scores are not clinically meaningful.

Interval morphine equivalent consumption did not show any significant difference between the TEA group and the TAP block group for 0–24 h, 24–48 h, and 48–72 h. However, we could obtain important results regarding the functional outcomes. The time to the first ambulation was significantly shorter in the TAP block group. Early ambulation is one of the important principles of ERAS, and previous studies have shown that early ambulation lowers the complication rate and reduces the patient's length of hospital stay^33^. The incidence of PONV and hypotension were also lower in the TAP block group compared to the TEA group.

Among the included studies in our meta-analysis, there were no studies documenting complications due to the intervention. There was no significant difference in the failure rate. However, in general, TAP block is regarded as a simple and safe technique. Among the reported complications are enlarged liver laceration, transient femoral nerve palsy, and bowel hematoma^34^, but the incidence can be further reduced by performing it under real-time ultrasound guidance. On the other hand, TEA requires caution because it has a higher risk of complications and may cause major complications such as epidural hemorrhage/hematoma, infection, and epidural abscess^35^.

We found significant heterogeneity regarding the surgery type of our included trials. A total of 12 RCTs were analyzed in our meta-analysis for the postoperative pain score at rest at 12 h, with 9 studies on open surgery, 2 studies on laparoscopic surgery, and 1 study on both open and laparoscopic surgery. Among the 16 studies analyzed for the postoperative pain score at rest at 24 h, 12 studies were on open surgery, 3 studies on laparoscopic surgery, and 1 study on both open and laparoscopic surgery. As laparoscopic surgeries are increasing and the intensity of postoperative pain could differ between open and laparoscopic surgery, more studies comparing the efficacy of TEA and TAP block in laparoscopic surgery are needed.

There have been previous meta-analyses regarding this issue^2,3,36^. In the most recent meta-analysis, Desai et al.^3^ reported a significant difference in the pain score at rest at 12 h with 11 RCTs favoring TEA, while our analysis shows no significant difference. For the pain score at rest at 24 h, there was no significant difference^3^. This may be due to a different number of studies included in the meta-analysis. Data collection was performed by direct contact with the authors in our analysis. However, TSA showed the same results favoring TEA for pain score at rest at 12 h. Hamid et al.^36^ published a meta-analysis with six RCTs only for colorectal surgery and reported that TAP block is equivalent to TEA regarding postoperative pain scores but provided better functional recovery with a lower incidence of complications. Our study also demonstrated that the time to ambulation was significantly shorter and the incidence of hypotension at 24 h was significantly lower in TAP block group compared to TEA group. Baeriswyl et al.^2^ analyzed 10 RCTs for both children and adults. There was no significant difference in their primary outcome of the pain score at rest at 24 h and they concluded that both techniques are equally effective for both children and adults. TAP block was associated with a fewer incidence of hypotension and reduced length of hospital stay.

Our meta-analysis has several limitations. Firstly, the risk of bias from individual studies is low. The quality of evidence for each outcome is low or very low. There was a high risk of performance bias and detection bias. Most studies did not have detailed descriptions of how they blinded participants, study personnel, and outcome assessor. Secondly, there is significant heterogeneity regarding the research methods of individual studies and the results of the meta-analysis for our study outcomes. The heterogeneous methods of TEA and TAP block administration, injection drugs, drug dose, catheter placement, and postoperative analgesia protocol after surgery make it difficult to pool the study results. Thirdly, for the comparison of hospital length of stay, the criteria for hospital discharge may vary in different institutions, which makes it difficult to compare TEA with TAP block groups. Also for the comparison of the incidence of hypotension, the different diagnostic criteria of hypotension undermine the validity of our results. Finally, we used estimated means and standard deviations from medians and interquartile ranges divided by 1.35. This method is valid only when the distribution of the outcome variable is similar to the normal distribution. As data on VAS score is frequently skewed, our estimation may lead to wrong estimation.

In conclusion, we could not find any significant or clinically meaningful difference in the postoperative pain scores until 72 h after surgery. Regarding pain scores, our meta-analysis may indicate that both techniques are equally effective. Our analysis demonstrated that time to ambulation was significantly shorter and the incidence of hypotension was significantly lower in the TAP block group compared to the TEA group. Regarding these outcomes, TAP block may be a better choice than TEA. However, TSA showed that the required information size has not yet been reached. Given the significant heterogeneity of our meta-analysis, high risk of bias of individual studies and low or very low quality of evidence for most of our outcomes, firm conclusions cannot be drawn but it is not likely that the addition of further studies could prove any clinically meaningful difference in the pain score between these two techniques.

## Supplementary Information

Supplementary Information.

## Data Availability

All other data is available in the Supplementary Information files. Any further information is available upon request from the corresponding author.

## References

[1] Zhang P, Deng X-Q, Zhang R, Zhu T (2015). Comparison of transversus abdominis plane block and epidural analgesia for pain relief after surgery. Br. J. Anaesth..

[2] Baeriswyl M, Zeiter F, Piubellini D, Kirkham KR, Albrecht E (2018). The analgesic efficacy of transverse abdominis plane block versus epidural analgesia: A systematic review with meta-analysis. Medicine.

[3] Desai N, El-Boghdadly K, Albrecht E (2021). Epidural vs. transversus abdominis plane block for abdominal surgery—A systematic review, meta-analysis and trial sequential analysis. Anaesthesia.

[4] Julian Higgins, J. T. Cochrane Handbook for Systematic Reviews of Interventions. https://training.cochrane.org/handbook/current (2021). Accessed 29 Nov 2022.

[5] Moher D, Liberati A, Tetzlaff J, Altman DG, PRISMA Group (2009). Preferred reporting items for systematic reviews and meta-analyses: The PRISMA statement. PLoS med..

[6] Higgins, J. P. T. & (Eds) G. S. Cochrane Handbook for Systemic Reviews of interventions (Version 5.1.0) [Updated March 2011]. The Cochrane Collaboration. www.cochrane-handbook.org (2011).

[7] Balshem H (2011). GRADE guidelines: 3. Rating the quality of evidence. J. Clin. Epidemiol..

[8] Higgins JP, Thompson SG (2002). Quantifying heterogeneity in a meta-analysis. Stat. Med..

[9] Higgins JP, Thompson SG, Deeks JJ, Altman DG (2003). Measuring inconsistency in meta-analyses. BMJ.

[10] Wetterslev J, Thorlund K, Brok J, Gluud C (2008). Trial sequential analysis may establish when firm evidence is reached in cumulative meta-analysis. J. Clin. Epidemiol..

[11] Aditianingsih D, Mochtar CA, Chandra S, Sukmono RB, Soamole IW (2018). Comparison of three-quadrant transversus abdominis plane block and continuous epidural block for postoperative analgesia after transperitoneal laparoscopic nephrectomy. Anesth. Pain Med..

[12] Calixto-Flores A, Diaz-Angulo W (2020). Effectiveness and safety of continuous transverse abdominal plane blocks vs. epidural analgesia in donor nephroureterectomy. Transplant. Proc..

[13] Canakci E, Gultekin A, Cebeci Z, Hanedan B, Kilinc A (2018). The analgesic efficacy of transverse abdominis plane block versus epidural block after caesarean delivery: Which one is effective? TAP block? Epidural block?. Pain Res. Manag..

[14] Cata JP (2021). The impact of thoracic epidural analgesia versus four quadrant transversus abdominis plane block on quality of recovery after cytoreductive surgery with hyperthermic intraperitoneal chemotherapy surgery: A single-center, noninferiority, randomized controlled trial. Ann. Surg. Oncol..

[15] Felling DR (2018). Liposomal bupivacaine transversus abdominis plane block versus epidural analgesia in a colon and rectal surgery enhanced recovery pathway: A randomized clinical trial. Dis. Colon Rectum.

[16] Ganapathy S (2015). Comparison of efficacy and safety of lateral-to-medial continuous transversus abdominis plane block with thoracic epidural analgesia in patients undergoing abdominal surgery: A randomised, open-label feasibility study. Eur. J. Anaesthesiol..

[17] Hughes MJ (2015). Randomized clinical trial of perioperative nerve block and continuous local anaesthetic infiltration via wound catheter versus epidural analgesia in open liver resection (LIVER 2 trial). Br. J. Surg..

[18] Kandi Y (2015). Efficacy of ultrasound-guided transversus abdominis plane block versus epidural analgesia in pain management following lower abdominal surgery. Ain-Shams J. Anaesthesiol..

[19] Iyer SS, Bavishi H, Mohan CV, Kaur N (2017). Comparison of epidural analgesia with transversus abdominis plane analgesia for postoperative pain relief in patients undergoing lower abdominal surgery: A prospective randomized study. Anesth. Essays Res..

[20] Mathew P (2019). Quality of recovery and analgesia after total abdominal hysterectomy under general anesthesia: A randomized controlled trial of TAP block vs. epidural analgesia vs. parenteral medications. J. Anaesthesiol. Clin. Pharmacol..

[21] Niraj G (2011). Comparison of analgesic efficacy of subcostal transversus abdominis plane blocks with epidural analgesia following upper abdominal surgery. Anaesthesia.

[22] Niraj G (2014). Comparison of analgesic efficacy of four-quadrant transversus abdominis plane (TAP) block and continuous posterior TAP analgesia with epidural analgesia in patients undergoing laparoscopic colorectal surgery: An open-label, randomised, non-inferiority trial. Anaesthesia.

[23] Raghvendra KP (2016). Postoperative pain relief following hysterectomy: A randomized controlled trial. J. Midlife Health.

[24] Rao Kadam V, Van Wijk RM, Moran JI, Miller D (2013). Epidural versus continuous transversus abdominis plane catheter technique for postoperative analgesia after abdominal surgery. Anaesth. Intensive Care.

[25] Regmi S (2019). Comparison of analgesic efficacy of continuous bilateral transversus abdominis plane catheter infusion with that of lumbar epidural for postoperative analgesia in patients undergoing lower abdominal surgeries. Indian J. Anaesth..

[26] Revie EJ, McKeown DW, Wilson JA, Garden OJ, Wigmore SJ (2012). Randomized clinical trial of local infiltration plus patient-controlled opiate analgesia vs. epidural analgesia following liver resection surgery. HPB.

[27] Shaker TM (2018). Efficacy and safety of transversus abdominis plane blocks versus thoracic epidural anesthesia in patients undergoing major abdominal oncologic resections: A prospective, randomized controlled trial. Am. J. Surg..

[28] Torgeson M, Kileny J, Pfeifer C, Narkiewicz L, Obi S (2018). Conventional epidural vs. transversus abdominis plane block with liposomal bupivacaine: A randomized trial in colorectal surgery. J. Am. Coll. Surg..

[29] Turan A (2022). Transversus abdominis plane block with liposomal bupivacaine versus continuous epidural analgesia for major abdominal surgery: The EXPLANE randomized trial. J. Clin. Anesth..

[30] Wahba SS, Kamal SM (2014). Analgesic efficacy and outcome of transversus-abdominis plane block versus low thoracic-epidural analgesia after laparotomy in ischemic heart disease patients. J. Anesth..

[31] Wu Y (2013). The analgesic efficacy of subcostal transversus abdominis plane block compared with thoracic epidural analgesia and intravenous opioid analgesia after radical gastrectomy. Anesth. Analg..

[32] Xu YJ (2020). Randomized clinical trial of continuous transversus abdominis plane block, epidural or patient-controlled analgesia for patients undergoing laparoscopic colorectal cancer surgery. Br. J. Surg..

[33] Varadhan KK (2010). The enhanced recovery after surgery (ERAS) pathway for patients undergoing major elective open colorectal surgery: A meta-analysis of randomized controlled trials. Clin. Nutr..

[34] Jankovic Z, Ahmad N, Ravishankar N, Archer F (2008). Transversus abdominis plane block: How safe is it?. Anesth. Analg..

[35] Freise H, Van Aken H (2011). Risks and benefits of thoracic epidural anaesthesia. Br. J. Anaesth..

[36] Hamid HKS, Marc-Hernandez A, Saber AA (2021). Transversus abdominis plane block versus thoracic epidural analgesia in colorectal surgery: A systematic review and meta-analysis. Langenbecks Arch. Surg..

