# Supplementary material for: Transverse abdominis plane block compared with patient-controlled epidural analgesia following abdominal surgery: a meta-analysis and trial sequential analysis
Source: Sci Rep. 2022 Nov 29;12:20606. doi: 10.1038/s41598-022-25073-w (PMC9709047; doi:10.1038/s41598-022-25073-w)
Supplement: Supplementary file 1 — Supplementary Information. [file 41598_2022_25073_MOESM1_ESM.docx]

**Transverse abdominis plane block compared with patient-controlled epidural analgesia following abdominal surgery – a meta-analysis and trial sequential analysis**

**: Supplemental Digital Content**

**Supplemental Figure S1**. Funnel plot of comparison: Pain score at rest at 12 h

**Supplemental Figure S2.** Funnel plot of comparison: Pain score at rest at 24 h

**Supplemental Table S1.** Results of the meta-analysis of the secondary outcomes.

**Supplemental Table S2**. Qualuty of the evidence (GRADE approach).

**Supplemental Figure S1**. Funnel plot of comparison: Pain score at rest at 12 h.

On the y-axis standard error of the mean difference of the outcome of interest (measure of trial size) was plotted as a function, on the x-aixs, of the mean difference of the outcome.


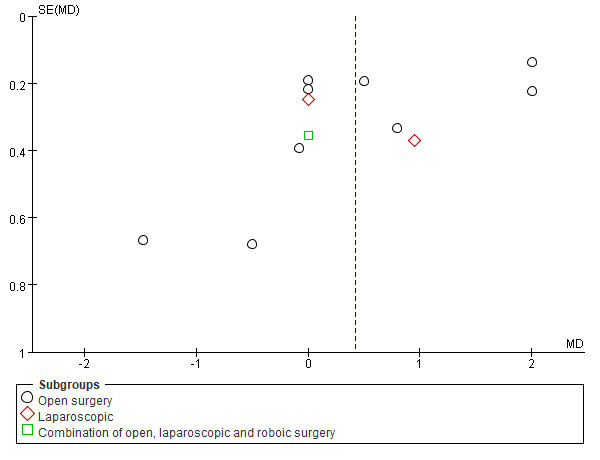


**Supplemental Figure S2**. Funnel plot of comparison: Pain score at rest at 24 h


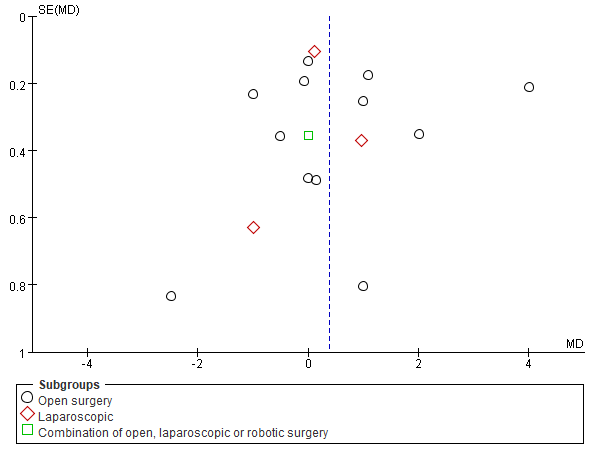


**Supplemental Table S1**. Results of the meta-analysis of the secondary outcomes.

| Outcomes | Number of studies included | TAP group | Epidural group | Effect size  (95% CI) | I^2^ (%) | P-value* |
| --- | --- | --- | --- | --- | --- | --- |
| Pain score at rest |  |  |  |  |  |  |
| at 0 – 2 h ^11,14,17,18,20,22-26,30-32^ | 13 | 413 | 404 | 0.46 (-0.17 to 1.08) | 94 | 0.15 |
| at 48 h ^14,16,17,21,22,24,26,27,30-32^ | 11 | 358 | 351 | 0.59 (0.15 to 1.03) | 86 | 0.009 |
| at 72 h ^16,17,21,24,29,31^ | 6 | 406 | 401 | 0.07 (-0.09 to 0.24) | 0 | 0.38 |
| Pain score on movement |  |  |  |  |  |  |
| at 0 – 2 h ^11,14,17,22-26,30-32^ | 11 | 308 | 299 | 0.79 (-0.10 to 1.68) | 93 | 0.08 |
| at 12 h ^11,16,17,21-23,25,26,30^ | 9 | 277 | 273 | 0.70 (-0.08 to 1.47) | 91 | 0.08 |
| at 24 h ^11,14,16,17,21-26,30-32^ | 13 | 416 | 409 | 0.86 (-0.42 to 2.13) | 98 | 0.19 |
| at 48 h ^14,17,21,22,24,26,30-32^ | 9 | 300 | 295 | 0.53 (0.07 to 0.99) | 76 | 0.03 |
| at 72 h ^16,17,21,24,31^ | 5 | 151 | 147 | -0.12 (-0.73 to 0.49) | 58 | 0.70 |
| Interval morphine equivalent consumption |  |  |  |  |  |  |
| at 0 – 24 h ^11,14-17,20,23-25,27,30,31^ | 12 | 415 | 403 | 3.01 (-3.55 to 9.58) | 96 | 0.37 |
| 24 – 48 h ^14-17,24,27,30^ | 7 | 278 | 264 | -15.62 (-34.70 to 3.46) | 98 | 0.11 |
| 48 – 72 h ^14-17,24,27,30^ | 7 | 278 | 264 | -1.05 (-5.33 to 3.24) | 85 | 0.63 |
| Postoperative clinical course |  |  |  |  |  |  |
| Time to first flatus (hours) ^15-17,22,28,30-32^ | 8 | 342 | 329 | 2.45 (-0.59 to 5.49) | 86 | 0.11 |
| Time to ambulation (hours) ^11,17,22,30,32^ | 5 | 181 | 177 | -4.52 (-8.68 to -0.36) | 70 | 0.03 |
| Hospital length of stay (days) ^14,15,18,22,24,26,28,32^ | 8 | 338 | 323 | -0.37 (-0.89 to 0.15) | 79 | 0.16 |
| Complication rate |  |  |  |  |  |  |
| Procedure failure rate ^11-19,21-26,28,31,32^ | 18 | 656 | 640 | 0.91 (0.48 to 1.72) | 0 | 0.76 |
| Incidence of nausea and vomiting ^18,20,28,31,32^ | 5 | 173 | 171 | 0.81 (0.39 to 1.65) | 50 | 0.55 |
| Incidence of hypotension at 24 h ^14,25,27^ | 3 | 102 | 103 | 0.30 (0.13 to 0.71) | 0 | 0.006 |
| Incidence of hypotension at 72 h ^16,17,31^ | 3 | 102 | 97 | 0.17 (0.06 to 0.48) | 0 | <0.001 |

The data are presented as mean difference or risk ratio with its 95% confidence interval (CI).

TAP = transversus abdominis plane

P-value is the result of the test for overall effect.

**Supplemental Table S2**. Qualuty of the evidence (GRADE approach).

|  | No. of studies | No. of TAP group | No. of Epidural group | Quality assessment | | | | | Quality of evidence |
| --- | --- | --- | --- | --- | --- | --- | --- | --- | --- |
|  |  |  |  | Risk of bias | Inconsistency | Indirectness | Imprecision | Publication bias |  |
| Pain score at rest |  |  |  |  |  |  |  |  |  |
| at 0-2h after surgery | 13 | 413 | 404 | Serious ^a^ | Serious ^b^ | Not serious | Not serious | Likely ^e^ | ⊕⊝⊝⊝ very low |
| at 12 h after surgery | 12 | 342 | 338 | Serious ^a^ | Moderate ^c^ | Not serious | Not serious | Likely ^e^ | ⊕⊕⊝⊝ Low |
| at 24 h after surgery | 15 | 486 | 479 | Serious ^a^ | Serious ^b^ | Not serious | Serious ^d^ | Likely ^e^ | ⊕⊝⊝⊝ very low |
| at 48 h after surgery | 11 | 358 | 351 | Serious ^a^ | Moderate ^c^ | Not serious | Not serious | Likely ^e^ | ⊕⊕⊝⊝ Low |
| at 72 h after surgery | 6 | 406 | 401 | Serious ^a^ | Not serious | Not serious | Not serious | Likely ^e^ | ⊕⊕⊝⊝ Low |
| Pain score on movement |  |  |  |  |  |  |  |  |  |
| at 0-2h after surgery | 11 | 308 | 299 | Serious ^a^ | Moderate ^c^ | Not serious | Not serious | Likely ^e^ | ⊕⊕⊝⊝ low |
| at 12 h after surgery | 10 | 277 | 273 | Serious ^a^ | Serious ^b^ | Not serious | Not serious | Unlikely | ⊕⊕⊝⊝ Low |
| at 24 h after surgery | 13 | 416 | 409 | Serious ^a^ | Serious ^b^ | Not serious | Not serious | Likely ^e^ | ⊕⊝⊝⊝ very low |
| at 48 h after surgery | 9 | 300 | 295 | Serious ^a^ | Serious ^b^ | Not serious | Not serious | Unlikely | ⊕⊕⊝⊝ Low |
| at 72 h after surgery | 5 | 151 | 147 | Serious ^a^ | Moderate ^c^ | Not serious | Not serious | Unlikely | ⊕⊕⊕⊝ Moderate |
| Inverval morphine equivalent consumption |  |  |  |  |  |  |  |  |  |
| at 0 – 24 h | 12 | 415 | 403 | Serious ^a^ | Serious ^b^ | Not serious | Not serious | Unlikely | ⊕⊕⊝⊝ Low |
| 24 – 48 h | 7 | 278 | 264 | Serious ^a^ | Serious ^b^ | Not serious | Not serious | Unlikely | ⊕⊕⊝⊝ Low |
| 48 – 72 h | 7 | 278 | 264 | Serious ^a^ | Serious ^b^ | Not serious | Serious ^d^ | Likely ^e^ | ⊕⊝⊝⊝ very low |
| Postoperative clinical course |  |  |  |  |  |  |  |  |  |
| Time to first flatus (hours) | 8 | 342 | 329 | Serious ^a^ | Serious ^b^ | Not serious | Not serious | Unlikely | ⊕⊕⊝⊝ Low |
| Time to ambulation (hours) | 5 | 181 | 177 | Serious ^a^ | Moderate ^c^ | Not serious | Not serious | Unlikely | ⊕⊕⊕⊝ Moderate |
| Hospital length of stay (days) | 8 | 338 | 323 | Serious ^a^ | Not serious | Not serious | Not serious | Unlikely | ⊕⊕⊕⊝ Moderate |
| Complication rate |  |  |  |  |  |  |  |  |  |
| Procedure failure rate | 18 | 656 | 640 | Serious ^a^ | Not serious | Not serious | Not serious | Unlikely | ⊕⊕⊕⊝ Moderate |
| Incidence of nausea and vomiting | 5 | 173 | 171 | Serious ^a^ | Not serious | Not serious | Serious ^d^ | Likely ^e^ | ⊕⊕⊝⊝ Low |
| Incidence of hypotension at 24 h | 3 | 102 | 103 | Serious ^a^ | Not serious | Not serious | Not serious | Too few studies | ⊕⊕⊝⊝ Low |
| Incidence of hypotension at 72 h | 3 | 102 | 97 | Serious ^a^ | Not serious | Not serious | Not serious | Too few studies | ⊕⊕⊝⊝ Low |

The basis for the assumed risk is provided in footnotes.

^a^ In most studies, bliding was not performed for participants, personnel and outcome assessors. Final decision to rate down quality of evidence by one level for risk of bias.

^b^ I^2^ was above 50% with wide variance of point estimates across studies. Final decision to rate down quality of evidence by one level for serious inconsistency.

^c^ Even though the I^2^ was above 50%, the point estimates did not vary widely between studies. Final decision to not rate down quality of evidence for moderate inconsistency.

^d^ Confidence interval included null effect as well as appreciable benefit and/or harm. Final decision to rate down quality of evidence by

one level for serious imprecision.

^e^ Final decision to rate down quality of evidence by one level for serious publication bias.

High quality means that we are very confident that the true effect lies close to that of the estimate of the effect. Moderate quality means that we are moderately confident in the effect estimate: The true effect is likely to be close to the estimate of the effect, but there is a possibility that it is substantially different. Low quality means that our confidence in the effect estimate is limited: The true effect may be substantially different from the estimate of the effect. Very low quality means that we have very little confidence in the effect estimate: The true effect is likely to be substantially different from the estimate of effect.
